# A novel regulator of selective autophagy: TNIP1/ABIN-1 modulates mitophagy

**DOI:** 10.1080/27694127.2023.2165269

**Published:** 2023-01-11

**Authors:** Rosetta Merline, Liliana Schaefer

**Affiliations:** Institute of Pharmacology and Toxicology, Goethe University, Frankfurt, Germany

**Keywords:** autophagy receptor, inflammation, LC3-interacting region, mitochondria, mitophagy

## Abstract

TNIP1/ABIN-1 is a novel inhibitor of inflammation and cell death. In our study, we elucidated the MAP1LC3/LC3 (microtubule associated protein 1 light chain 3)-interacting region (LIR) 1 and 2 motifs of TNIP1-dependent direct binding to LC3B-II and subsequent colocalization on phagophores. In addition, TNIP1 colocalizes with LAMP1 (lysosomal associated membrane protein 1) on autolysosomes. Autophagic stimuli induce the translocation of TNIP1 to damaged mitochondria promoting the degradation of the mitochondrial outer membrane proteins VDAC1 (voltage dependent anion channel 1), MFN2 (mitofusin 2), and TOMM20 (translocase of outer mitochondrial membrane 20), together with its own degradation, possibly via the autophagic pathway. Knockdown of TNIP1 partially but significantly inhibits mitophagy. Thus, identification of TNIP1 as a novel selective mitophagy regulator promoting mitophagy would play a decisive role in understanding the pathophysiological outcome associated with diverse types of mitochondria-associated cellular stress.

Mitochondria, apart from being the powerhouse of the cells, is an organelle associated with a plethora of functions, including apoptosis. Mitochondrial integrity plays a vital role and is decisive in regulating cellular survival and death. Mitophagy is a selective autophagic process regulating mitochondrial turnover to maintain cellular integrity and homeostasis. It involves sequestration of damaged mitochondria by selective autophagy receptors (SARs) mediated by either the PINK1 (PTEN induced kinase 1)-PRKN/parkin regulated ubiquitin-dependent pathway or ubiquitin-independent receptor-mediated pathway into *de novo*-formed autophagosomes and subsequent autolysosomal degradation. Selective mitophagy receptors are the best studied among the SARs and a number of them have been identified including BCL2 (BCL2 apoptosis regulator), BNIP3 (BCL2 interacting protein 3), BNIP3L/NIX (BCL2 interacting protein 3 like), FUNDC1 (FUN14 domain containing 1), SPATA33 (spermatogenesis associated 33), FKBP8 (FKBP prolyl isomerase 8), and PHB2 (prohibitin 2). Dysregulated mitophagy alters mitochondrial homeostasis and disrupts the delicate balance between selective autophagy and cellular apoptosis. Thus, elucidation of molecular mechanisms regulating mitophagy would help decipher the pathologies associated with diverse inflammatory diseases.

TNIP1/ABIN-1, genetically encoded on chromosome 5q32-33.1, is a polyubiquitin-binding ubiquitously expressed protein located in the cytoplasm and nuclear compartments of the cell. Structurally, TNIP1 has four ABIN homology domains (AHD 1-4) of which AHD1 binds TNFAIP3/A20, while AHD2 binds polyubiquitin and polyubiquitinated proteins. The C terminus of TNIP1 has an IKBKG/NEMO binding domain (NBD). TNIP1 plays a vital role in regulating diverse cellular functions. The role of TNIP1 in regulating cell death is elucidated based on its ability to suppress apoptosis and necroptosis. Its role in autophagy became evident based on the regulation of ubiquitination and/or proteasome/lysozyme??-dependent degradation of HDAC1 (histone deacetylase 1), TAX1BP1 (Tax1 binding protein 1), ARRB2 (arrestin beta 2), and NLRP10. TNIP1 shares high sequence homology with the SAR OPTN (optineurin). TNIP1 was recently identified as an anti-inflammatory signal-induced autophagy receptor that attenuates NFKB activation. Accordingly, TNIP1 has therapeutic implications in treating several inflammatory diseases, including colitis, systemic lupus erythematosus, sepsis, glomerulonephritis, and psoriasis to mention a few. In our recent manuscript [1], we aimed to elucidate the role of TNIP1 as a novel regulator of mitophagy and delineated the key functions of its MAP1LC3/LC3 (microtubule associated protein 1 light chain 3)-interacting region (LIR) domains in the process.

Selective autophagy relies on SAR-mediated recognition and delivery of cargo to lipidated phagophore-bound LC3 through LIRs. Our results confirmed the binding of TNIP1 with LC3B via its LIR motifs. Co-immunoprecipitation assays with hemagglutinin (HA)-tagged full-length (FL) TNIP1 and TNIP1 variants with point mutations in LIR1, LIR2, or both (LIR1+2) in transfected HeLa cells revealed predominant binding of TNIP1-HA FL protein with the membrane-associated LC3B-II, rather than the cytosolic LC3B-I. Mutation in LIR2 significantly inhibits the binding compared to that of mutated LIR1, whereas mutation of both LIR domains (LIR1+2) completely inhibits the binding suggesting preferential binding of LIR2 over LIR1 to LC3B. Using glutathione S-transferase (GST) affinity-isolation experiments with purified bacterial TNIP1-FL and LIR-mutant proteins, we proved the LIR-dependent direct binding of TNIP1 to LC3A and LC3B, indicating a role of TNIP1 as a SAR associated with LC3B-II on the membrane of phagophores.

TNIP1, similar to the SQSTM1 (sequestosome 1)-like selective mitophagy receptors is structurally endowed with multimerization capabilities, LIR-motifs, and ubiquitin-binding domain/UBD, required for the execution of mitophagy. TBK1 (TANK binding kinase 1)-mediated phosphorylation of TNIP1 induces its selective autophagy-dependent degradation. In our study, induction of autophagy in doxycycline-inducible monomeric green fluorescent protein (mEGFP)-TNIP1 and mCherry-LC3B co-transfected HeLa cells results in colocalization of mEGFP-TNIP1 with mCherry-LC3B-positive vesicles, clustering with LAMP1-containing lysosomes, and degradation via autophagic flux, suggesting its regulation similar to that of a SAR via the autophagic pathway.

Mitophagy is orchestrated by mitochondrially recruited SARs including BNIP3L, BNIP3, FUNDC1, AMBRA1 (autophagy and beclin 1 regulator 1), FKBP8, PHB2, NIPSNAP1, and NIPSNAP2. In the present study, activation of mitophagy enhances the expression of TNIP1 in the mitochondrial fraction compared to that of its restricted expression in the cytosolic fraction in unstimulated mCherry-PRKN-expressing HeLa cells, suggesting its mitophagy-induced translocation to damaged mitochondria and a probable role as a selective mitophagy receptor. Ubiquitin-dependent mitophagy promotes ubiquitination of specific outer mitochondrial membrane proteins for proteasomal degradation. In the present study, immunoblotting revealed inhibition of mitophagy-induced degradation of mitochondrial proteins VDAC1, MFN2, and TOMM20 in clustered regularly interspaced short palindromic repeats (CRISPR)-CRISPR-associated protein 9 (Cas9)-mediated deleted *TNIP1* knockout compared to that of HeLa-mCherry-PRKN cells, suggesting TNIP1-dependent degradation of mitophagy substrates. Our results support a previously undescribed function of TNIP1 as a selective mitophagy receptor/adapter deciphering its resemblance to SARs in specifically targeting mitochondria as a specific cargo to phagophores for degradation. Ectopic targeting of TNIP1 to mitochondria induces mitophagy. In our study, fluorescence-activated cell sorting/FACS analysis reveals partial inhibition of mitophagy in small interfering RNA (siRNA)-*TNIP1*-treated compared to siRNA control PRKN-expressing mt-mKEIMA-cells. The limited inhibition of mitophagy in the absence of TNIP1 might be attributed to compensation by other SARs based on their ability to bind ubiquitin and to recruit to the same cargo. Further studies are required to elucidate the specific role of TNIP1 either as a SAR or as an adaptor protein in mitophagy.

Thus, our study identified TNIP1 as a putative SAR/adaptor recruited to damaged mitochondria. Through its LIR-dependent binding to LC3B-II and colocalization with autolysosomes, TNIP1 is co-degraded as a mitophagy SAR/adapter together with mitochondrial outer membrane proteins along the autophagy pathway promoting mitophagy.

To date, TNIP1 has been targeted in treating inflammation-associated auto-immune diseases. Considering the myriad of cellular processes regulated by mitochondria including cell death, the deciphered role of TNIP1 as a modulator of mitophagy would help us understand the complex cellular machinery regulating mitochondrial homeostasis, the complex molecular crosstalk involved in mitochondria-associated regulation of inflammation and diverse cell death fates, and in the development of therapeutics for treating mitochondria-associated pathologies.
